# A Minimally Invasive Surgical Technique for Hallux Valgus Deformity Correction Using an Ultrasonic Bone Scalpel (All India Institute of Medical Sciences Technique)

**DOI:** 10.7759/cureus.31524

**Published:** 2022-11-15

**Authors:** Vijay Kumar Digge, Nitin Chauhan, Aritra Chattopadhyay, Sai Krishna MLV

**Affiliations:** 1 Orthopaedics, All India Institute of Medical Sciences, New Delhi, New Delhi, IND

**Keywords:** piezoelectric, bone scalpel, deformity, minimally invasive surgery, hallux valgus

## Abstract

Hallux valgus is a common forefoot deformity characterized by medial deviation of the first metatarsal and lateral deviation of the hallux. More than 150 procedures have been described for the hallux valgus deformity with no proven superiority of one over the other. The initial osteotomies are open, and with the advent of power and micro instruments, the osteotomies were manageable via mini incisions and percutaneous procedures. The minimally invasive procedures have been divided into three generations. The first-generation osteotomies involve wedge correction. The second and third-generation osteotomies are translational. The second generation is a simple osteotomy, and the third is a chevron-type osteotomy. In our technique, we have used a hybrid procedure of second and third-generation procedures. The technique uses an ultrasonic bone scalpel to create a transverse sub-capital osteotomy which is then fixed with screws for a stable construct.

## Introduction

Hallux valgus is one of the most common deformities of the lower extremity, with an estimated prevalence of 23% in adults between 18 to 65 years and 35.7% in adults over the age of 65 years [1]. The surgical procedures for the hallux valgus deformity correction date back to the nineteenth century, and the procedure involved resection of the head of the metatarsal. It was Reverdin in 1881 who first described an open, closed wedge osteotomy at the sub-capital level of the metatarsal [1,2]. The osteotomies for the deformity correction can be done either at the metaphyseal level of both proximal and distal or at the diaphyseal level. The distal metaphyseal osteotomies were described by Hohmann, Wilson, Mitchell, and Chevron. The proximal metaphyseal osteotomies were described by Ludloff, Mann, and Chevron. The diaphyseal osteotomy was scarf osteotomy [1-4]. The level of osteotomies is also classified based on the amount of surgical exposure. The initial osteotomies are open, and with the advent of power instruments and micro instruments, the osteotomies were manageable via mini incisions and percutaneous procedures. The percutaneous procedures are performed through the skin with stab incisions. The minimally invasive procedures are those between the open and percutaneous procedures [1-4]. In this surgical technique, we described a minimally invasive procedure for hallux valgus deformity correction with percutaneous screw fixation without bone loss, preventing metatarsal shortening.

## Technical report

**Figure 1:**
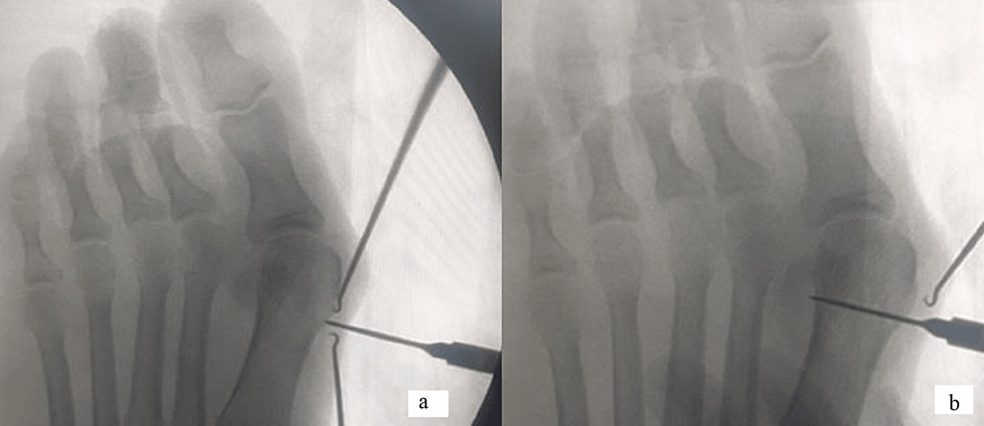
(a and b) Sub-capital osteotomy being performed with an ultrasonic bone scalpel.

The patient was placed supine on a radiolucent table with a tourniquet over the thigh. The patient is operated on under regional anesthesia. The knee is supported with a bolster so that the hip and knee are flexed with the foot in plantigrade and parallel to the floor. The surgery was performed under image guidance. The specific instrumentation required for the surgery is an ultrasonic bone scalpel (BoneScalpel; Misonix, Farmingdale, NY), 3.5 mm and 4.0 mm headless cannulated screws (FT Screw, Arthrex), and 4.0 mm cannulated cancellous screws. The ultrasonic bone scalpel consists of a console, handpiece, footplate, and tubings. The console is set to standard setting with amplitude at 7, pulse at 100%, and flow at 70%. The surgical procedure planned was a minimally invasive distal metaphyseal transverse osteotomy with an ultrasonic bone scalpel and percutaneous screw fixation, the AIIMS technique. First, a one-centimeter skin incision is made on the medial side of the distal metaphyseal region of the first metatarsal. This is followed by subcutaneous dissection, and once the bone is reached, the position is confirmed under image guidance (Figure 1a). Next, a transverse osteotomy is performed with an ultrasonic bone scalpel (Figure 1b).

**Figure 2:**
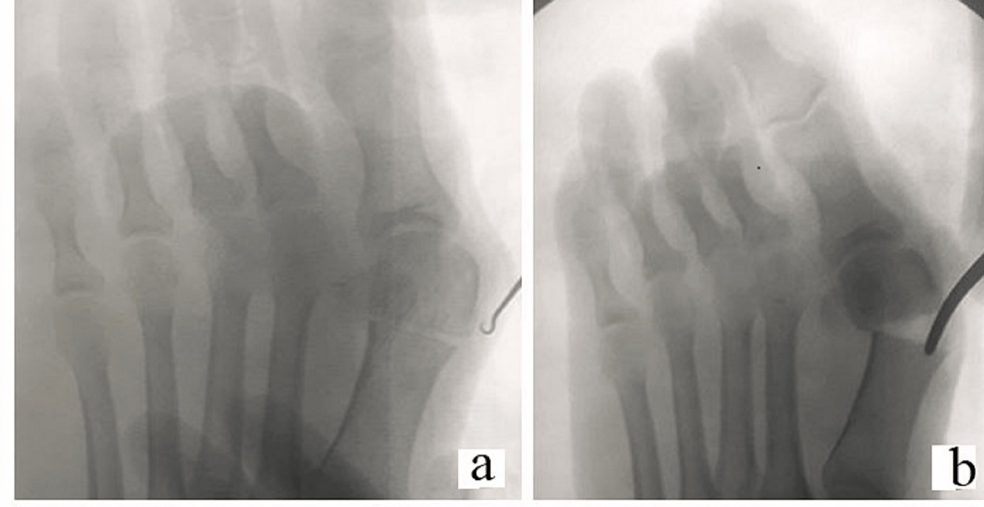
(a and b) After complete osteotomy, the deformity is corrected by displacement of the fragments.

Once the osteotomy is completed (Figure 2a), the proximal shaft is displaced laterally under image guidance (Figure 2b).

**Figure 3:**
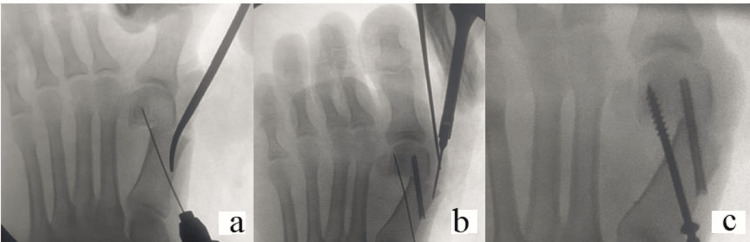
(a and c) The osteotomy site after displacement and deformity correction is fixed with screws. (b) The bony prominence after deformity correction is shaved off.

The osteotomy site is fixed with either headless screws (3.5 mm FT screw, Arthrex) or 4 mm cannulated cancellous screws. Percutaneously also, the prominent dorsomedial border of the displaced proximal shaft is shaved off (Figure 3). 

**Figure 4:**
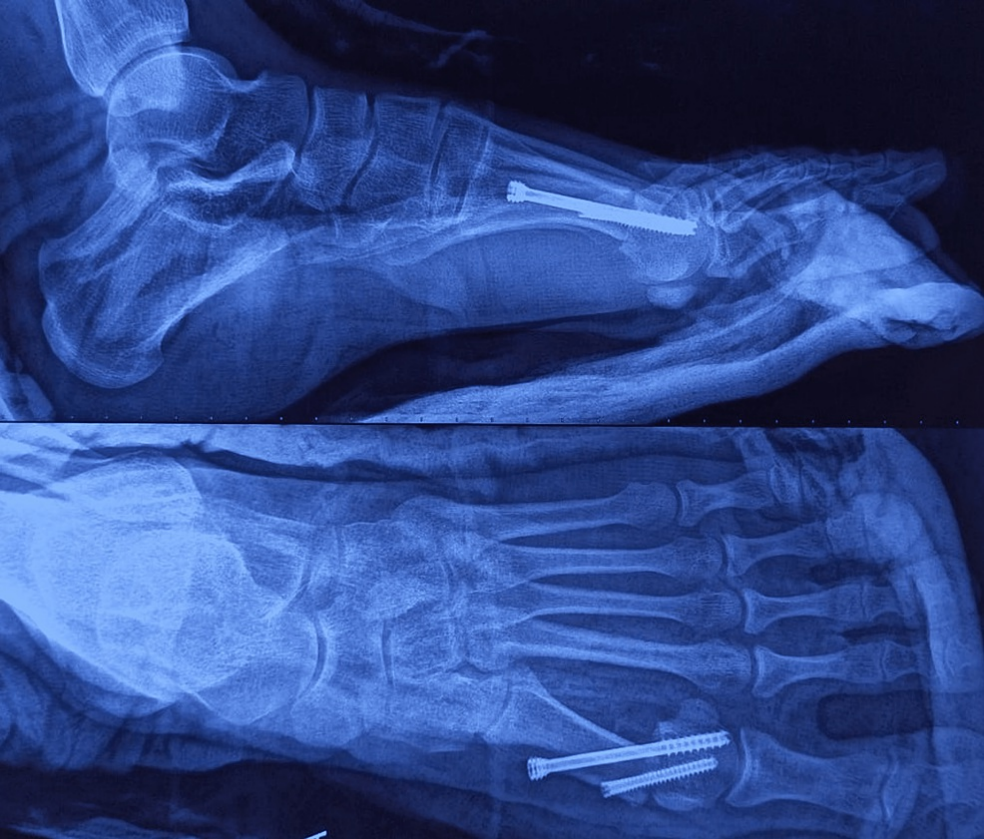
Post-operative radiograph.

**Figure 5:**
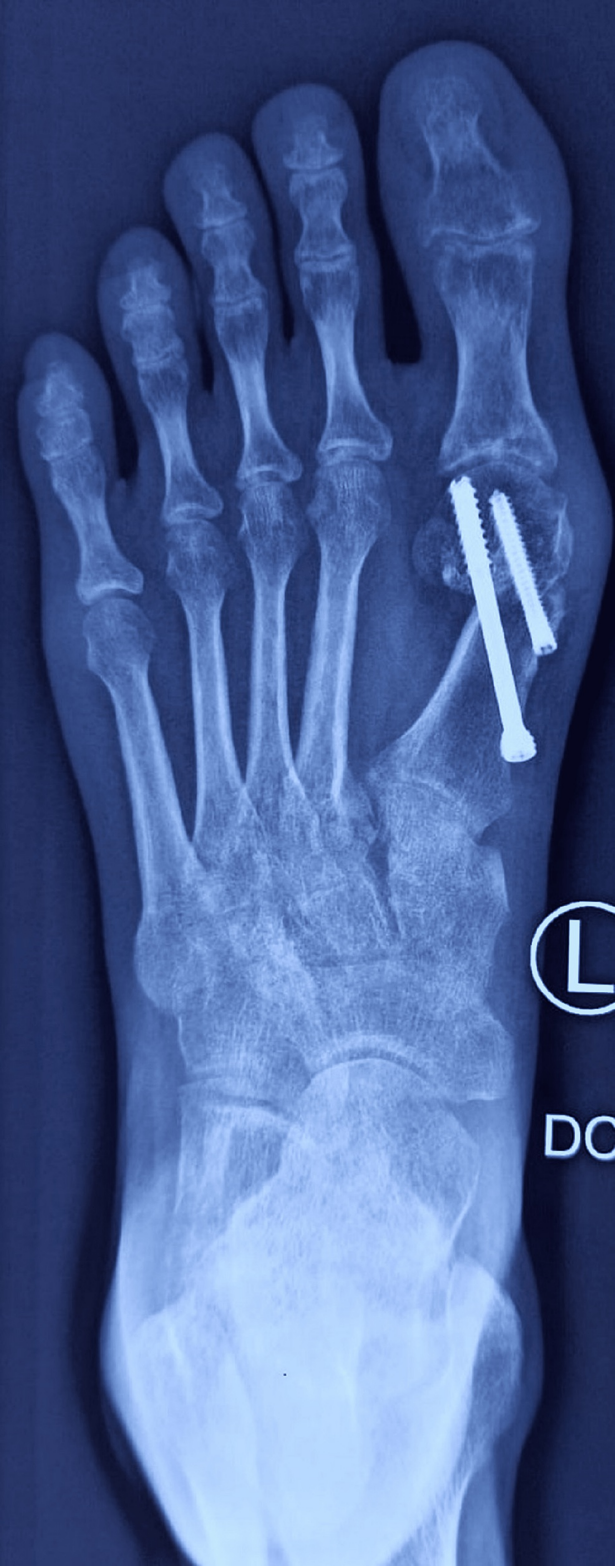
Post-operative radiograph at three months.

The reduction is checked under the image, and if required, Akin osteotomy is performed at the proximal phalanx. The wound is closed, and a below-knee plaster is applied for two weeks. The suture removal was done at two weeks. The patient is started on ankle and toes range of movement at two weeks, partial weight bearing at six weeks, and progressing to full weight bearing by three months. The immediate postoperative radiograph showed maintenance of alignment (Figure 4), and postoperative radiographs at three months showed consolidation and maintenance of alignment (Figure 5).

## Discussion

Hallux valgus is a common forefoot deformity characterized by medial deviation of the first metatarsal and lateral deviation of the hallux. More than 150 procedures have been described for the hallux valgus deformity with no proven superiority of one over the other. Open osteotomies, which were the initial surgeries, are associated with complications like scar tenderness, risk of infection, and osteonecrosis of the head. With the advent of microsurgical instruments, minimally invasive and percutaneous surgery have become popular. Percutaneous surgery involves stab incisions, and the minimally invasive technique involves smaller incisions of up to 1 cm. Till now, three generations of minimally invasive procedures have been described [4].

The first-generation osteotomy is the reproduction of the Reverdin procedure in a minimally invasive technique using micro instruments. The first-generation technique is the Isham procedure, which is a closing wedge incomplete intraarticular osteotomy at the distal metaphyseal level done using a 2 mm burr and does not involve any implants for fixation [1,2].

The second-generation osteotomies are Bosch and Endolog techniques, considered a modification of the Hohmann procedure. These procedures involve transverse sub-capital osteotomies done at the distal metaphyseal level using a 2 mm burr. The proximal fragment was displaced medially and held with an intramedullary K-wire or an intramedullary device [1,2].

The third-generation osteotomies are similar to second-generation but involve a stable internal fixation at the osteotomy site. The third-generation procedures are described by Redfern and Vernois and involve a chevron osteotomy at the distal metaphyseal level rather than a simple transverse sub-capital osteotomy of the second generation. After the osteotomy, the proximal fragment is displaced medially, just like in the second generation, but the fragments were fixed with headless screws in a percutaneous fashion [1,2].

The first-generation minimally invasive procedures are closing wedge osteotomies, resulting in metatarsal shortening. First-generation osteotomies do not involve any implants. The second and third-generation osteotomies are translational procedures whereas the osteotomy is transverse in the second generation and a chevron type in the third generation. Though there is a risk of non-union in translational osteotomies, the available literature suggests that 30% of cortical contact is adequate to prevent non-union. Hence up to 70% of translation can be done in second and third-generation osteotomies to correct the deformity. In second-generation procedures, the fixation across osteotomy is not done, and there is a risk of pin site infections and malunions [3].

All three generations of minimally invasive procedures use a 2 mm burr for the osteotomy. In the first generation, there is a higher risk of metatarsal shortening due to the removal of a wedge of bone. In the second and third generations also, using a 2 mm burr to make either a transverse or chevron osteotomy results in metatarsal shortening, which could cause metatarsalgia in the long term [4].

Piezoelectric and ultrasonic vibrations have been used to cut tissues for the last 30 years, and recently their use has been extended to cutting bone, mostly in spinal surgeries. The clinical advantages of piezosurgery are due to its selective cutting, precision, and low-temperature work environments. The piezoelectric systems convert electric current into mechanical vibrations and, at an ultrasonic frequency, are used to cut the bone. The other advantage being at an ultrasonic speed, they selectively cut mineralized tissues only. Unfortunately, only a hand full of studies used the piezoelectric saw for osteotomy purposes in hallux valgus [5]. In our technique, we have used a bone scalpel instead of a saw which works at ultrasonic frequency using piezoelectric technology. In addition, we have used a second-generation osteotomy technique and implants of the third generation.

## Conclusions

The benefits of our technique for doing osteotomy are that it is a minimally invasive procedure, it is a transverse osteotomy that can be performed at ease rather than a biplanar chevron osteotomy, and stable fixation with screws which addresses the problem of malunion and non-union. The other advantages are those of the ultrasonic bone scalpel, which does not cause bone loss and resultant metatarsal shortening, and the disadvantage is the cost of the bone scalpel. The soft tissues are also protected while osteotomy with an ultrasonic scalpel, which is not the case with burrs and saws.
